# The Neuroprotective Effects of *Oroxylum indicum* Extract in SHSY-5Y Neuronal Cells by Upregulating BDNF Gene Expression under LPS Induced Inflammation

**DOI:** 10.3390/nu16121887

**Published:** 2024-06-14

**Authors:** Shareena Sreedharan, Alpana Pande, Anurag Pande, Muhammed Majeed, Javier Villela-Castrejon, Luis Cisneros-Zevallos

**Affiliations:** 1 Department of Horticultural Sciences, Texas A&M University, College Station, TX 77843, USA; shareenasreedharan@gmail.com (S.S.); jav.cas@tamu.edu (J.V.-C.); 2 Analytical R&D Department, Sabinsa Corporation, East Windsor, NJ 08520, USA; alpana@sabinsa.com (A.P.); anurag@sabinsa.com (A.P.); mmajeed@sabinsa.com (M.M.); 3 Department of Food Science & Technology, Texas A&M University, College Station, TX 77843, USA

**Keywords:** oroxylin A, chrysin, baicalein, brain-derived neurotrophic factor (BDNF), neuro inflammation, LPS, *Oroxylum indicum*, SHSY-5Y neuronal cells

## Abstract

The brain-derived neurotrophic factor (BDNF) plays a crucial role during neuronal development as well as during differentiation and synaptogenesis. They are important proteins present in the brain that support neuronal health and protect the neurons from detrimental signals. The results from the present study suggest BDNF expression can be increase up to ~8-fold by treating the neuroblastoma cells SHSY-5Y with an herbal extract of *Oroxylum indicum* (50 μg/mL) and ~5.5-fold under lipopolysaccharides (LPS)-induced inflammation conditions. The *Oroxylum indicum* extract (Sabroxy) was standardized to 10% oroxylin A, 6% chrysin, and 15% baicalein. In addition, Sabroxy has shown to possess antioxidant activity that could decrease the damage caused by the exacerbation of radicals during neurodegeneration. A mode of action of over expression of BDNF with and without inflammation is proposed for the *Oroxylum indicum* extract, where the three major hydroxyflavones exert their effects through additive or synergistic effects via five possible targets including GABA, Adenoside A2A and estrogen receptor bindings, anti-inflammatory effects, and reduced mitochondrial ROS production.

## 1. Introduction

The brain-derived neurotrophic factor (BDNF) is a member of the neurotrophin family [1] that plays a prominent role in the central nervous system (CNS) function such as neuronal development, differentiation, synaptogenesis, neural protection from harmful stimuli, managing long-term memory [2], and shielding from neuronal degeneration [3]. Abnormal or decreased regulation of BDNF may affect CNS function. Furthermore, low levels of BDNF have been associated to neurological disorders including Alzheimer’s and Parkinson’s disease [4,5,6]. *Oroxylum indicum* is a promising plant with the potential to increase BDNF levels due to the presence of flavonoids that have shown neuroprotective effects. The major flavones constituents in *Oroxylum indicum* include oroxylin A (OA) a natural O-methylated flavone, chrysin (CH) a 5,7-dihydroxyflavone, and baicalein (BA) a 5,6,7-trihydroxyflavone [7,8]. These chemical compounds have been reported to exert neuroprotective effects and possess anti-inflammatory and antioxidant properties [9,10,11]. For instance, previous studies have highlighted the importance of OA in modulating brain-derived neurotrophic factor (BDNF) function [12,13]. The *Oroxylum indicum* extract can be derived from the bark of *Oroxylum indicum* tree, also known as the Indian trumpet tree, and can be optimized or standardized to 10% OA, 6% CH, and 15% BA (Sabroxy extract).

A recent study has shown that the standardized *Oroxylum indicum* extract (Sabroxy) can exert antioxidant activity during chemotherapy-treated mice and also, it can enhance the mitochondrial function in the brain cells [14], and in clinical studies, it has been shown to improve cognitive function in older adults with self-reported cognitive complaints [8]; nevertheless, more studies are necessary to elucidate the detailed mechanism involved in its beneficial effects. The objective of this work was to study the effects of an herbal extract of *Oroxylum inducum* containing OA, CH, and BA on the BDNF expression using differentiated human neuroblastoma SH-SY5Y cells with and without inflammation conditions stimulated by LPS as a relevant human cell model that can be associated to clinical studies. We hypothesize that a standardized OA, CH, and BA-containing supplements based on *Oroxylum indicum* could be useful in treating such neurological disorder conditions by increasing the production of BDNF.

## 2. Materials and Methods

A validated extract of *Oroxylum indicum* tree was obtained from solvent extraction of the bark of the tree. The dried bark was cut and pulverized into a fine to coarse powder and extracted with aqueous alcohol. The resultant extract was further purified using appropriate solvent. After removing the solvent, the dried extract was investigated for active constituents using spectroscopic techniques and later standardized using flavonoid constituents occurring naturally in the *Oroxylum indicum* extract (Sabroxy). The details are available in US patent 10555.982 [15].

### 2.1. HPLC-MS Analysis

Chromatographic analysis of Sabroxy was carried out using the previously published HPLC method and the US patent US10555982B2 [15,16], using Agilent 1200 series (Agilent Technologies, Waldbronn, Germany). A gradient mobile phase consisting of 0.1% formic acid in water (A) and acetonitrile (B) was used in the analysis at a flow rate of 0.9 mL/min. The gradient used was as follows: 0 → 40 min, 30% B; 40 → 50 min, 40% B; 50 → 55 min, 50% B; 55 → 65 min, 100% B, 65 → 70 min, 70% B. The chromatograms were recorded at 268 nm wavelength using a photodiode array detector. The sample was analyzed using Luna C-18 (250 mm × 4.6 mm, 5 µm) column (Phenomenex, Torrance, CA, USA). Peak identification and quantification of baicalein (BA), chrysin (CH) and oroxylin A (OA) was based on retention times of the standards. The Mass spectra was recorded using Agilent 6120 single quadrupole MS coupled with HPLC. MS system conditions were as follows: capillary voltage 3000 V, drying gas flow, 12 L/min, nebulizer pressure, 35 psi, and drying gas temperature of 350 °C. The MS data was generated using the ESI mode and ChemStation software version (C.01.07.(27)).

Sample preparation was performed in methanol (155 mg/50 mL) and filtered using PTFE (Polytetrafluoroethylene) 0.45 µm filter before injecting (20 µL) into the HPLC system.

### 2.2. Cell Culture

SH-SY5Y cells were grown on Corning 100-mm tissue culture dishes (Corning, NY, USA) in DMEM/F12 media supplemented with 10% Heat inactivated (HI)-FBS, 100 units/mL penicillin, and 100 μg/mL streptomycin with incubation at 37 °C in humidified 5% CO_2_ chamber. Cells were differentiated using DMEM/F12 with 10 μM RA, 100 units/mL penicillin, 100 μg/mL streptomycin, and changing the HI-FBS supplementation to 1% for 10 days. The medium was changed every other day during differentiation.

### 2.3. Cell Viability Assay

Cells were plated at 5 × 10^3^ cells/well in a 96-well clear bottom black plate (Costar, Cambridge, MA, USA), after 24 h post-seeding, the cells were treated with 10 µg/mL, 20 µg/mL, and 50 µg/mL of Sabroxy extract dissolved in DMSO for another 24 h. Cell viability was measured using CellTiter 96^®^ AQueous One Solution Cell Proliferation Assay (Promega Corporation, Madison, WI, USA) according to the manufacturer’s instructions. The quantity of formazan product was measured at 490 nm and is directly proportional to the mitochondrial dehydrogenase activity. The cell viability results are expressed as percentages normalized to untreated cells.

### 2.4. LPS-Induced Inflammation

To evaluate the effect of Sabroxy extract against LPS exposure, differentiated cells were plated at 5 × 10^5^ cells/well in a 6-well plate (BD Biosciences, Franklin Lakes, NJ, USA), and treated with the growth medium containing 10 µg/mL LPS with a 5 h pre-treatment of Sabroxy extracts using a preventive model approach in this study. RNA extraction was performed with the RNeasy Mini Kit (74104 Qiagen, Hilden, Germany); RNA samples were collected and stored at −80 °C until further use.

### 2.5. qPCR

Enzyme reverse transcriptase (High-Capacity cDNA reverse transcription kit 4368814. Applied Biosystems, Waltham, MA, USA) was used to prepare cDNA from RNA, qPCR was performed using the primers shown in Table 1.

### 2.6. Reactive Oxygen Species (ROS) Levels

ROS status was evaluated using DCFH-DA assay according to Martinez et al. [17], with slight modifications. After treatment with Sabroxy extracts and LPS, cells were washed twice with PBS and incubated for 1 h with 10 µM DCFH in serum-free media, washed again twice with PBS, and the fluorescence intensity was measured at 485 nm for excitation and 528 nm for emission using a plate reader (Synergy HT, Bio-Tek Instruments, Inc., Winooski, VT, USA).

### 2.7. Cellular Antioxidant Activity (CAA) Assay

100 µL of a cell suspension of 6 × 10^5^ differentiated cells were plated in 96-well clear bottom black plates. After 24 h, the cells were washed with PBS and exposed to several concentrations of Sabroxy in free-serum DMEM/F12 with a final concentration of 10 µM of DCFHDA and incubated for 30 min. The cells were washed three times with PBS, and then 100 µL of AAPH at 500 µM was added. The generated fluorescence due to radical generation was monitored for 1.5 h every 2 min at 485 nm for excitation and 528 nm for emission using a plate reader (Synergy HT, Bio-Tek Instruments, Inc., Winooski, VT, USA). The area under the curve (*AUC*) was integrated and the CAA unit was calculated as follows:

$$\mathrm{CAA}\;\mathrm{Unit}=\left(1-\frac{{AUC}_{Sample}}{{AUC}_{Control}}\right)\times 100$$

where *AUC_Sample_* is the integrated area under the sample and *AUC_control_* is the control without any treatment [18].

### 2.8. ORAC Assay

Chemical Antioxidant Activity was measured using the oxygen radical absorbance capacity (ORAC) assay as previously reported by Thaipong et al., [19]. Briefly, the ORAC procedure used an automated plate reader (KC4, Bio-Tek Instruments, Inc., Winooski, VT, USA) with clear bottom 96-well black plates. Assays were conducted in phosphate buffer pH 7.4 at 37 °C. Peroxyl radical was generated using 2,2′-azobis (2-amidino-propane) dihydrochloride that was prepared fresh for each run. Fluorescein was used as the substrate. Fluorescence conditions were as follows: excitation at 485 nm and emission at 520 nm. The standard curve was linear between 0 and 50 μM Trolox. Results were expressed as µM Trolox equivalents (TE)/mg of the sample.

### 2.9. Statistical Analysis

Results are expressed as means ± standard errors (SE) from the three biological repeats. One-way analysis of variance (ANOVA) followed by Tukey HSD as well as t-student analysis was used to test for differences among the treatment groups using the software JMP Pro v10.0. Different letters show significant differences at *p* ≤ 0.05.

## 3. Results

### 3.1. HPLC-MS Analysis of Oroxylum indicum Extract

**Figure 1:**
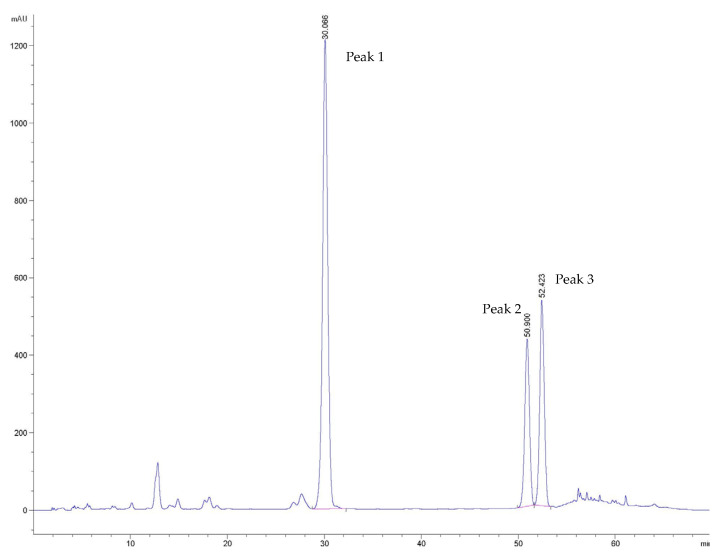
HPLC chromatogram of a standardized *Oroxylum indicum* methanolic extract (Sabroxy).

The HPLC analysis of the Sabroxy sample, using the method previously developed by Majeed et al. [15], showed no interference from co-eluters. As observed in the chromatograms, major peaks eluted between retention times (Rt) of 10–34 min and 48–55 min (Figure 1, Table 2), which were further selected for studies by MS spectroscopy. Both positive and negative ESI modes were used for identification.

**Figure 2:**
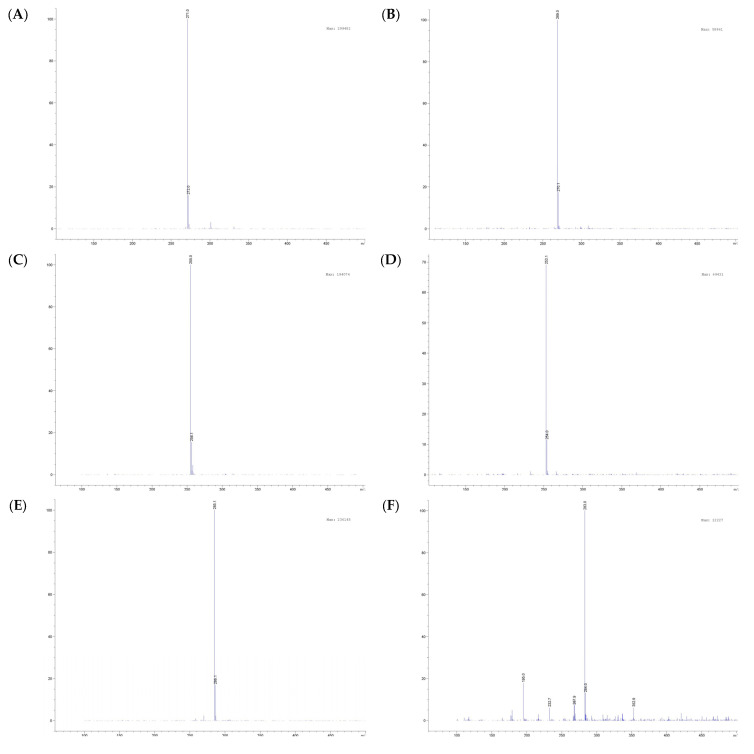
Mass spectrum analysis of a standardized *Oroxylum indicum* extract (Sabroxy). Mass spectrum of (**A**,**B**) peak 1—baicalein; (**C**,**D**) peak 2—chrysin; and (**E**,**F**) peak 3—Oroxylin A in ESI positive and ESI negative, respectively.

Based on the HPLC separation and Mass determination, compounds identified in the *Oroxylum indicum* extract (Sabroxy^®^) were baicalein (Peak 1 Rt 30.066 min; Figure 2A,B), chrysin (Peak 2 Rt 50.900 min; Figure 2C,D) and oroxylin-A (Peak 3 Rt 52.423 min; Figure 2E,F) with a content of ~15%, 6%, and 10%, respectively (Table 2).

### 3.2. Viability of SHSY-5Y Cells Treated with Oroxylum indicum Extracts

**Figure 3:**
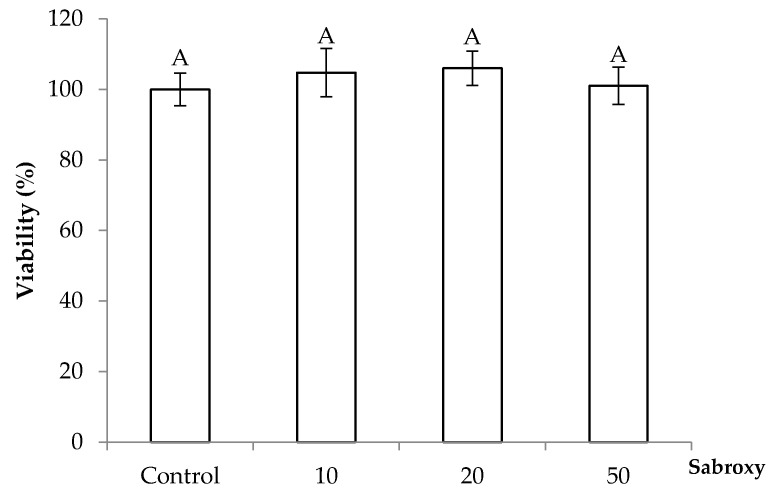
Cell viability of SHSY-5Y neural cells after treatment with a standardized *Oroxylum indicum* extract (Sabroxy containing 10% OA, 6% CH, 15% BA) was determined by the MTS assay. Assays were performed on 3 replicates for each treatment. Viability measured by MTS is expressed as the percentage of controls at 24 h treatment. Data are expressed as mean ± standard errors (SE). One-way analysis of variance (ANOVA) followed by Tukey HSD showing values with a different letter(s) are statistically different (*p* ≤ 0.05).

The cell viability results for the Sabroxy extract are shown in Figure 3. A 10 µg/mL, 20 µg/mL, and 50 µg/mL of the extract corresponding to ~1, 2, and 5 µg/mL of OA, ~0.6, 1.2, and 3 µg/mL of CH, and ~1.5, 3, and 7.5 µg/mL of BA, respectively, were non-cytotoxic to differentiated SHSY-5Y cells (*p* ≥ 0.05). Similar results were obtained by Liu et al. [20] when they were working with OA in microglial BV-2 cells up to almost 29 µg/mL (100 µM) without any reduction in cell viability.

### 3.3. BDNF Expression in SHSY-5Y Cells Treated with Oroxylum indicum Extracts

**Figure 4:**
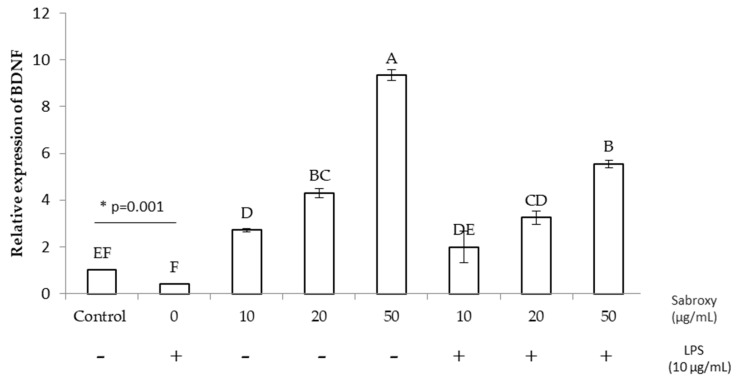
The effect of a standardized *Oroxylum indicum* extract (Sabroxy) on BDNF gene expression in SHSY-5Y neural cell with and without inflammation conditions. The gene expression analysis was performed by RT-PCR, using differentiated SHSY-5Y neural cells. BDNF expression increases with 10, 20, and 50 µg/mL Sabroxy in the presence and absence of inflammation induced by LPS (10 µg/mL). The gene transcripts were normalized using β-actin as a control. Data, obtained from n = 6 repeats at least, are shown as mean ± SE. One-way analysis of variance (ANOVA) followed by Tukey HSD showing values with a different letter(s) are statistically different (*p* < 0.05). * t-student analysis showed significant difference between controls and LPS treated cells at *p* = 0.001.

To investigate whether *Oroxylum indicum* extracts affects BDNF expression, we evaluated gene expression using differentiated SHSY-5Y cells treated with Sabroxy. The results showed that BDNF levels were increased by *Oroxylum indicum* extracts in a concentration-dependent manner up to 8-fold increase with 50 µg/mL of the extract compared to the control (Figure 4, *p* ≤ 0.05). The same trend was obtained when cells were challenged with LPS and 5 h of pretreatment with Sabroxy. Interestingly, even with the addition of LPS induced inflammation, Sabroxy extracts were able to restore BDNF expression to basal levels and even enhance it up to 5.5-fold when treated with 50 µg/mL of Sabroxy (*p* ≤ 0.05). Furthermore, LPS downregulated the BDNF expression in control SHSY-5Y cells by ~60% due to an induced inflammation state (t-student, *p* = 0.001).

### 3.4. ROS Levels in SHSY-5Y Cells Treated with Oroxylum indicum Extracts

**Figure 5:**
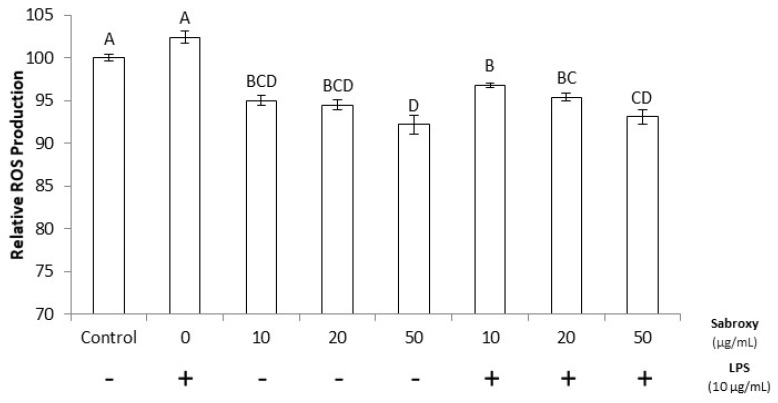
Effect of a standardized *Oroxylum indicum* extract (Sabroxy) in LPS-induced ROS expression status in SH-SY5Y neural cells after 19 h of incubation, using DCFHDA as a reporter. Data represent the mean ± SE of six independent experiments in triplicate. One-way analysis of variance (ANOVA) followed by Tukey HSD showing values with a different letter(s) are statistically different (*p* ≤ 0.05).

Several studies have linked oxidative stress to the presence of LPS [21]; thus, we determined the ROS status at the end of the cell treatments (Figure 5). After 19 h of LPS incubation, the ROS levels of the control (No LPS) versus those of LPS alone did not differ statistically (*p* ≥ 0.05); however, there was a trend with the addition of Sabroxy where the ROS status was lower than the basal levels (*p* ≤ 0.05). Similar results were obtained by Eren et al., [22] with the addition of sulforaphane on N9 microglial cells also stimulated with LPS.

### 3.5. Antioxidant Activity of Oroxylum indicum Extract Evaluated by the CAA and ORAC Assays

**Figure 6:**
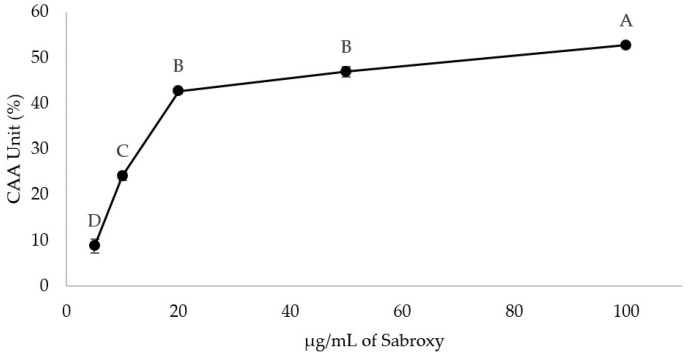
Cellular Antioxidant Activity of different concentrations of a standardized *Oroxylum indicum* extract (Sabroxy) in differentiated SH-SY5Y neural cells. Cells were exposed to DCFCH-DA and Sabroxy extracts, then the ROS generator AAPH was added, and the reaction was monitored for 1.5 h. Data represent the mean ± SE of six independent experiments in triplicate. One-way analysis of variance (ANOVA) followed by Tukey HSD showing values with a different letter(s) are statistically different (*p* ≤ 0.05).

To study the efficacy of Sabroxy extract against ROS, we performed the CAA assay using AAPH as a radical generator (Figure 6). This assay is intended to measure the ability of compounds to prevent the transformation of DCFHDA from being oxidized by endogen radicals or a radical generator; the CAA takes into consideration the cellular uptake and metabolism of cells. Each CAA unit represents a percent in the reduction of fluorescence in comparison with the radical generator [23]. The results showed an increase in the CAA units with Sabroxy extracts with concentrations ranging from 5 µg/mL to 100 µg/mL with a maximum inhibition of ROS of 52.7% with 100 µg/mL, proving that the Sabroxy extract can be internalized in a short time (30 min of incubation) and can decrease ROS levels (*p* ≤ 0.05). There was a marked increase of CAA units, from 5–20 µg/mL with a lower augment of CAA units from the following concentrations, reaching nearly a plateau curve at 50 µg/mL. In addition, we performed the ORAC assay, which measures the ability of a substance to inhibit the peroxyl-radical induced oxidation. The result showed an ORAC value of 8.42 ± 0.24 µM TE/mg of Sabroxy indicating a high level of antioxidant activity similar to that of other supplements like resveratrol (8.6 µM TE/mg), green tea (13.814 µM TE/mg) [24], and Santa herba (8.19 µM/mg) [25].

## 4. Discussion

LPS has been used as a tool to understand the mechanisms during neurodegeneration [26]. The expression of inflammatory mediators upon activation of the TLR4 receptor by LPS is one of the main contributors of the degenerative process; the pathway of such pro-inflammatory molecules as TNF-α and IL-β1 downregulate the levels of neurotrophins, a series of growth factors for the development of neurons. One well-studied neurotrophin is BDNF, whose importance relies on that it participates in numerous pathways like plasticity, growth, and neuronal survival [27]. Thus, is highly important to restore the balance of neurotrophins as a therapeutic strategy [28]. In this work, we evaluated the use of a natural extract derived from *Oroxylum indicum* containing a standardized 10% of OA, 6% CH and 15% BA (Sabroxy) as a potent inductor of BDNF on SH-SY5Y cells with neuronal phenotype. When cells were exposed to Sabroxy, the viability was not affected at the highest concentration tested 50 µg/mL (~5, 3 and 7.5 µg/mL of OA, CH, and BA, respectively), demonstrating the feasibility for the use of Sabroxy. This extract has also been used in mice in a sub-acute study with doses of 500 mg/kg (~50, 30 and 75 mg/kg of OA, CH and BA, respectively) without any hepatotoxic effect [29], and more recently in clinical studies on cognitive function in older adults receiving daily 500 mg of Sabroxy extract twice (~100, 60, and 150 mg of OA, CH, and BA daily, respectively) [8]. In addition, pharmacokinetics studies by Fong et al., [30] showed that the oral administration of 15 mg/kg of OA in mice resulted in a metabolization to baicalein and then glucuronidation to become oroxyloside. However, the authors also found a rapid increase of OA in the brain in a concentration around 1307 pmol/g, proving that when administered orally, OA can go through the blood–brain barrier and target neurons.

Since the standardized *Oroxylum indicum* extract (Sabroxy), is mostly a mix of three hydroxyflavones, namely chrysin (5,7-dihydroxyflavone, ~6%), baicalein (5,6,7-trihydroxyflavone, ~15%), and oroxylin A (5,7-dihydroxy-6-methoxyflavone, ~10%), at the maximum concentration used in this study of 50 μg/mL, this represents a mix of 9 μM of chrysin, 27 μM of baicalein, and 17 μM of oroxylin. The amounts present for each compound could have specific targets and favor specific pathway routes in SH-SY5Y cells used in the present study. Furthermore, these compounds within the *Oroxylum indicum* extract could exert additive or synergistic neuroprotective effects during BDNF upregulation under scenarios where the SH-SY5Y cells are challenged with or without inflammation.

**Figure 7:**
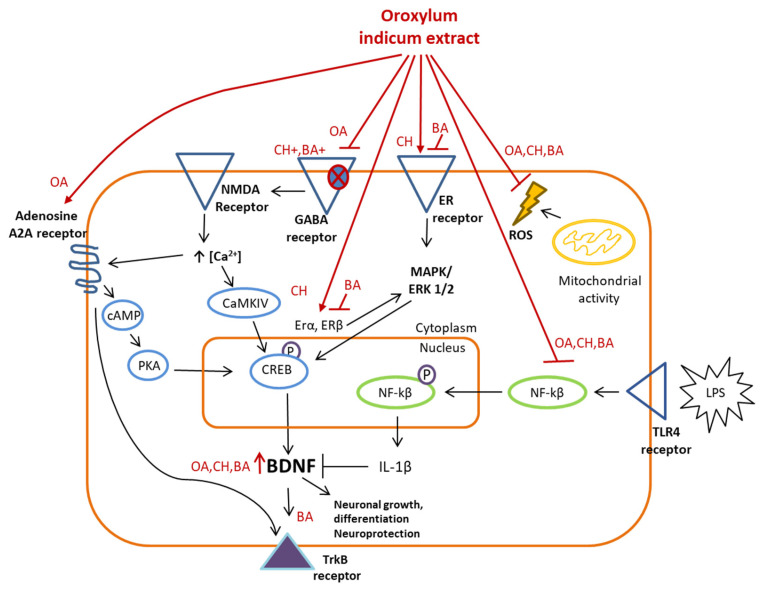
Proposed mechanisms of action of a standardized *Oroxylum indicum* extract (Sabroxy) in SH-SY5Y neural cells with or without inflammation. Active components of the extract like oroxylin A (OA), chrysin (CH), or baicalein (BA) elicit upregulation of BDNF through five possible targets. Namely, OA binds to the GABA receptor and blocks its expression resulting in activation of the NMDA receptor that turns out in a sustained increase of intracellular calcium levels that will lead to the activation of two pathways. One related to Calcium/calmodulin-dependent protein kinase IV (CaMKIV) and the other related to the Adenosine A2A receptor and the cAMP/PAK-MAPK pathway. Both routes will lead to the phosphorylation of cyclic AMP response element binding protein (CREB) and the subsequent BDNF expression. CH and BA may exert positive allosteric modulation of GABA receptor enhancing the OA antagonist effect. The activation of the A2A receptor will also transactivate TrkB, which initiates the TrkB-Akt pathway for neuronal survival. In addition, CH may activate estrogen receptors, while BA may attenuate this effect. Furthermore, all three compounds in the *Oroxylum indicum* extract exhibit free-radical scavenging activities that could reduce the reactive oxygen species (ROS) produced by the mitochondria as well as anti-inflammatory properties by attenuating NF-kβ and reducing pro-inflammatory cytokine levels. In the proposed model LPS induces inflammation through activation of pNF-kβ, where cytokine IL-1β suppresses BDNF expression. A + signal indicates positive allosteric modulation of receptor. Adapted from: [12,31,32,33,36,37,38,39].

Accordingly, a proposed mode of action of the standardized *Oroxylum indicum* extract could be based on previously reported effects exerted by the individual compounds and their possible interactions, as shown in Figure 7. In the following section, we discuss the proposed model of the neuroprotective effect by the Sabroxy extract based on reported individual activities of OA, CH, and BA.

Jeon et al., [12] and Bae et al. [31] proposed a mechanism by which OA could exert a neuroprotective effect by enhancing BDNF levels in neuronal cells via activation of CREB through GABA and Adenosine A2A receptors by antagonist and agonist properties, respectively. Thus, in Figure 7, the proposed mode of action for OA within the *Oroxylum indicum* extract in SH-SY5Y cells, based on the available literature and the present results, suggests that OA would inhibit directly GABA receptor and activate an influx of calcium through NMDA receptors. This increase in calcium will trigger two pathways, the first one related to Calcium/calmodulin-dependent protein kinase IV (CaMKIV), and the second one related to activation of Adenosine A2A receptor and the cAMP/PAK-MAPK pathway [32,33]. Both routes will lead to cAMP response element-binding protein (CREB) phosphorylation and the subsequent expression of BDNF in the nucleus. In addition, OA could also directly activate the Adenosine A2A receptor and trigger the expression of BDNF [31].

The present study showed that the Sabroxy extract increases the BDNF expression levels in SH-SY5Y cells with or without an LPS-induced inflammation state, with a boost in BDNF levels of 5.5-fold and 8- fold increase compared to basal levels, respectively. This calls attention since in other studies, some phytochemicals are only able to restore the basal level of BDNF expression [34,35].

In addition to in vivo studies of OA showing neuroprotective effects by increase in BDNF and CREB levels, other reports have shown that OA possess anti-inflammatory properties in vitro by reduction of pNF-kβ and cytokines IL-1β [7,40].

For instance, Ye et al., [38] studied the role of OA during inflammation studies in RAW264.7 cells. OA can upregulate nuclear factor erythroid 2-related factor 2 (Nrf2) and antioxidant response elements (ARE) for increased anti-inflammatory properties. This can be translated into a reduction of pro-inflammatory genes (iNOS and COX-2), which has also been associated to a suppression of NF-kβ by OA [41]. Another neuroprotective effect of OA was studied by Ji et al., [13] with a model using amyloid-beta (Aβ) peptide as inductor of inflammation in PC12 cells; OA ranging from 10–500 µM was able to downregulate the NF-kβ pathway with a reduction of inflammatory cytokines (TNF- α, NO and PGE2), probably due to a lower expression of the above-mentioned mechanisms. In the present study, LPS treatment influenced BDNF expression without altering oxidative stress, meaning that the reduction in BDNF was not related to a disbalance in ROS status but rather other mechanisms, likely a direct effect of LPS/TLR4 receptor on increased levels of NF-kβ. It has been found that SH-SY5Y cells express TLR4 in very low concentrations [42], supporting the possibility of another pathway by which LPS can affect neurons and their survival during the progression of neuro-diseases. For instance, it has been proposed that the inflammatory cytokine IL-1β can disrupt BDNF signaling cascades [43] as well as decrease its expression [36].

During neuroinflammation, other elicitors except LPS can increase ROS levels due to several factors, including an accumulation of Aβ peptide, metal toxicity, or a dysfunction of the mitochondria [13,26,44], in where the Sabroxy containing OA could exert an important antioxidant effect, as shown in the CAA assay. It is important to consider that other phytochemicals reported in Sabroxy, like baicalein, chrysin, and some glycosylated derivatives [15], could play key roles as antioxidants as well as neuroprotectors [45,46].

For instance, chrysin has shown antioxidant properties in vivo studies by increasing GHS, CAT, and anti-inflammatory effects by reducing NF-kβ, NO, and cytokines IL-1β levels. Chrysin shows neurotrophic effects and can modulate estrogen receptors (ERα and ERβ of membrane) triggering the MAPK/ERK1/2 signaling pathway involved in phosphorylation and subsequent CREB activation promoting the increase of BDNF levels in mice. In vitro studies have shown reduction in ROS formation and decrease in NO, PGE2, and TNF-α biosynthesis as well as attenuation of IL-1β and NF-kβ expression. In addition, chrysin allosterically modulates the GABA receptor through the benzodiazepine-binding site, both in vitro and in vivo studies [47,48].

On the other hand, baicalein and its glucuronide form, baicalin, have shown, in in vivo studies to increase BDNF expression, as well as pCREB/CREB protein ratios, reduce inflammation through attenuating NF-kβ-p65 and inflammatory cytokines IL-1β, as well as anti-oxidant properties by decreasing ROS levels [49]. Similarly, studies in mice have shown recovery of BDNF levels by attenuating inflammatory cytokines [50]. In general, baicalin shows antioxidant and anti-inflammatory properties in vitro and in vivo models [51]. Furthermore, baicalein and baicalin are positive allosteric modulators of GABA-A receptors in the benzodiazepine site and/or a non-benzodiazepine site [51,52,53], and is antagonist of estrogen receptors [54,55].

In addition, to the above properties, baicalein activates the BDNF/TrkB/CREB pathway in mice exerting neuroprotective effects [56,57] and/or alternatively activating the BDNF/TrkB receptor and exerting effects through the cAMP/pERK and PI3K/pAKT signaling [58] and increasing further the production of BDNF.

Based on the individual effects of CH and BA described above, the proposed model for the neuroprotective effects of these compounds within the *Oroxylum indicum* extract (Figure 7) would also include the activation of estrogen receptors and the MAPK-ERk1/2- CREB pathway to upregulate BDNF. Furthermore, CH and BA would act as positive allosteric modulators of GABA-A receptors to enhance the antagonistic effects of OA in the *Oroxylum indicum* extract. This proposed interaction would have to be confirm in further studies.

Allosteric modulators change the receptor conformation, bind to a receptor at a site different from the primary active site, altering its sensitivity or response to the ligand, agonist and antagonist binding [59]. For instance, the GABA-A receptor response, when stimulated by GABA, can be enhanced by allosteric modulators like benzodiazepines. However, the effects of allosteric modulators on ligands, agonist, and antagonists might not necessarily equally alter their affinity and efficacy on the receptor. Thus, the efficacy or affinity of the ligand on the receptor may be altered while not that of the agonist or antagonist, or the opposite effects as well [60,61].

In summary, we hypothesize that the three major compounds OA, CH, and BA present within the *Oroxylum indicum* extract could favor five specific targets. These include GABA receptor binding (OA as antagonist, and CH and BA as allosteric modulators), Adenosine A2A receptor binding (OA as agonist), estrogen receptors bindings (CH as agonist and BA as antagonist), attenuating pro-inflammatory transcription factor NF-kβ and inflammatory cytokines (all three compounds), or neutralizing oxygen based free radicals (all three compounds). These possible five specific targets of the standardized *Oroxylum indicum* extract would imply a complex regulation of pathways for BDNF biosynthesis with cross-talks among them deriving in additive or synergistic effects elicited by the three major compounds.

For instance, if additive effects predominate, then all five targets might be activated individually, where the overall final effect in BDNF increase levels is the result of adding each individual response. However, if synergistic effects predominate, then it is likely it would take place through the enhancement of the antagonistic effects of OA on the GABA receptor by the positive allosteric modulation of CH and BA. Furthermore, even partial inhibitory or attenuating effects could take place at the estrogen receptor level depending on the affinity or efficacy of agonist CH or antagonist BA on the receptor. Further studies are need in vitro and in vivo to elucidate the specific roles of each compound in the observed BDNF overexpression of the *Oroxylum indicum* extract with and without inflammation and its neuroprotective properties. In addition, in neurodegenerative diseases like Alzheimer’s Disease, the inhibitory and excitatory ratio is changed; thus, the need to understand how the extract may affect these ratios through the possible mode of actions described above.

Herein, we used a preventive model; thus, therapeutic effects should be explored as well. The present study can set the basis for those future studies.

## 5. Conclusions

In the present study, we found that an *Oroxylum indicum* extract standardized to a 10% OA, 6% CH, and 15% BA (Sabroxy extract) enhances BDNF expression in a dose-dependent manner in the presence and absence of neuronal inflammation induced by LPS. The protective role of the extract may be potentially relevant for instance under exacerbated inflammation led by immune senescence during aging. Furthermore, the preventive model used herein suggests exploring the extract at an early age in future clinical studies

Despite that the *Oroxylum indicum* extract having antioxidant properties and may reduce ROS levels, ROS does not seem to mediate the inflammation response in SH-SY5Y cells. Accordingly, we propose that *Oroxylum indicum* extract overcomes the negative effects of inflammation by exerting an increased overexpression of BDNF. Further studies are needed to elucidate if *Oroxylum indicum* extract has a direct effect in reducing inflammation in neuronal SH-SY5Y cells. We hypothesize that the bioactive flavones present in the standardized *Oroxylum indicum* extract exert their effect by additive or synergistic effects through five possible targets of the individual compounds including binding to GABA, Adenosine A2A, and estrogen receptors, and through anti-inflammatory and anti-oxidant activities. Further studies are needed to elucidate the contribution of each component in the neuroprotective effects of the extract and their ability to go through the blood–brain barrier and target neurons.

Decreased levels of BDNF have been detected in patients with neurological issues. Interestingly, various studies have also demonstrated that rebuilding of BDNF signaling liberates various symptoms in an animal model with neurological disturbance. Thus, Sabroxy may be useful for the induction of BDNF levels. Further preclinical models are encouraged to study the specific role of the three major flavone compounds present in the *Oroxylum indicum* extract, as well as to determine the appropriate dose for future clinical studies.

## Figures and Tables

**Table 1:** Primer sequences for real-time PCR assays.

Gene	Primer Sequences
BDNF	Forward: 5′-AGCTGAGCGTGTGTGACAGTATTAG-3′
	Reverse: 5′-ATTGCTTCAGTTGGCCTTTTGATAC-3′
β-Actin	Forward: 5′-CCCAGGCATTGCTGACAGG-3′
	Reverse: 5′-TGGAAGGTGGACAGTGAGGC-3′

**Table 2:** Phytochemical identification of a standardized *Oroxylum indicum* extract (Sabroxy) sample with their masses as identified by LC-MS.

Peak No.	Rt (min)	UV, λmax	Content (*w*/*w*)	ESI-MS (*m*/*z*)	Name of Identified Compounds
1	30.06	276,324	15	ESI − 269 ESI + 271	Baicalein (5,6,7-trihydroxyflavone)
2	50.9	268,314	6	ESI − 253 ESI + 255	Chrysin (5,7-dihydroxyflavone)
3	52.4	272,318	10.18	ESI − 283 ESI + 285	Oroxylin A (5,7-dihydroxy-6-methoxyflavone)

## Data Availability

Data are contained within the article.
