# Correction: Sreedharan et al. The Neuroprotective Effects of *Oroxylum indicum* Extract in SHSY-5Y Neuronal Cells by Upregulating BDNF Gene Expression Under LPS Induced Inflammation. *Nutrients* 2024, *16*, 1887

**DOI:** 10.3390/nu18081303

**Published:** 2026-04-21

**Authors:** Shareena Sreedharan, Alpana Pande, Anurag Pande, Muhammed Majeed, Javier Villela-Castrejon, Luis Cisneros-Zevallos

**Affiliations:** 1 Department of Horticultural Sciences, Texas A&M University, College Station, TX 77843, USA; 2 Analytical R&D Department, Sabinsa Corporation, East Windsor, NJ 08520, USA; 3 Department of Food Science & Technology, Texas A&M University, College Station, TX 77843, USA

## Addition of an Author

Javier Villela-Castrejon was not included as an author in the original publication [1]. The corrected Author Contributions statement appears as: “Conceptualization, L.C.-Z., A.P. (Alpana Pande) and A.P. (Anurag Pande); methodology, S.S., A.P. (Alpana Pande), J.V.-C.; formal analysis, S.S., J.V.-C.; investigation, S.S., J.V.-C.; resources, L.C.-Z. and M.M.; data curation, S.S.; writing—original draft preparation, S.S.; writing—review and editing, S.S., A.P. (Anurag Pande) and L.C.-Z.; funding acquisition, M.M. and L.C.-Z.; supervision, L.C.-Z.; project administration, L.C.-Z.; All authors have read and agreed to the published version of the manuscript.”

## Funding

In the original publication, the Funding statement “This research received a monetary gift from Sabinsa Corporation. In addition, it was supported in part by funding from the Texas A&M AgriLife Institute for Advancing Health through Agriculture (IHA)” was not completely included [1].

## Acknowledgments

In the original publication the Acknowledgements statement “We acknowledge the technical support of Vimal Nair.” was not completely included [1].

## Conflicts of Interest

In the original publication the Conflict of Interest statement “M.M, Al.P. and An.P., are paid employees of Sabinsa Corporation. The remaining authors declare that the research was conducted in the absence of any commercial or financial relationships that could be construed as a potential conflict of interest. The authors declare that this study received a monetary gift from Sabinsa Corporation. The funder was not involved in the study design, collection, analysis, interpretation of data, the writing of this article or the decision to submit it for publication.” was not completely included [1].

The authors state that the scientific conclusions are unaffected. This correction was approved by the Academic Editor. The original publication has also been updated.

## References

[1] Sreedharan S., Pande A., Pande A., Majeed M., Villela-Castrejon J., Cisneros-Zevallos L. (2024). The Neuroprotective Effects of *Oroxylum indicum* Extract in SHSY-5Y Neuronal Cells by Upregulating BDNF Gene Expression under LPS Induced Inflammation. Nutrients.

